# Lack of robustness of textural measures obtained from 3D brain tumor MRIs impose a need for standardization

**DOI:** 10.1371/journal.pone.0178843

**Published:** 2017-06-06

**Authors:** David Molina, Julián Pérez-Beteta, Alicia Martínez-González, Juan Martino, Carlos Velasquez, Estanislao Arana, Víctor M. Pérez-García

**Affiliations:** 1 Mathematical Oncology Laboratory (MÔLAB), Universidad de Castilla-La Mancha, Ciudad Real, Spain; 2 Neurosurgery Department, Hospital Universitario Marqués de Valdecilla and Fundación Instituto de Investigación Marqués de Valdecilla, Santander, Spain; 3 Radiology Department, Fundación Instituto Valenciano de Oncología, Valencia, Spain; George Washington University, UNITED STATES

## Abstract

**Purpose:**

Textural measures have been widely explored as imaging biomarkers in cancer. However, their robustness under dynamic range and spatial resolution changes in brain 3D magnetic resonance images (MRI) has not been assessed. The aim of this work was to study potential variations of textural measures due to changes in MRI protocols.

**Materials and methods:**

Twenty patients harboring glioblastoma with pretreatment 3D T1-weighted MRIs were included in the study. Four different spatial resolution combinations and three dynamic ranges were studied for each patient. Sixteen three-dimensional textural heterogeneity measures were computed for each patient and configuration including co-occurrence matrices (CM) features and run-length matrices (RLM) features. The coefficient of variation was used to assess the robustness of the measures in two series of experiments corresponding to (i) changing the dynamic range and (ii) changing the matrix size.

**Results:**

No textural measures were robust under dynamic range changes. Entropy was the only textural feature robust under spatial resolution changes (coefficient of variation under 10% in all cases).

**Conclusion:**

Textural measures of three-dimensional brain tumor images are not robust neither under dynamic range nor under matrix size changes. Standards should be harmonized to use textural features as imaging biomarkers in radiomic-based studies. The implications of this work go beyond the specific tumor type studied here and pose the need for standardization in textural feature calculation of oncological images.

## Introduction

Textural analysis refers to a variety of mathematical methods used to quantify the spatial variations in grey levels within an image to derive the so-called ‘textural features’. These techniques have attracted much attention recently because of their potential use as imaging biomarkers [1], in part because of their connection with the concept of tumoral heterogeneity [2, 3]. Also, the emerging field of radiomics has used textural features, among other imaging features, as information proxies for characterizing tumors [4, 5, 6].

Many methods have been proposed to quantify tumor texture and heterogeneity from imaging data. First-order features quantify the grey level distribution accounting for the frequency of appearance of each grey level within the tumor [7]. Second-order textural features construct grey level relations between pairs of voxels. Co-occurrence matrix (CM) features [8] are one of the most used type of second order method.

On the other hand, run-length matrix (RLM) based features quantify the heterogeneity by measuring the distributions and area sizes (groups of connected voxels) within the tumor having similar grey level values, providing information on regional heterogeneity [9].

Texture characterization using CM and RLM-based methods has been extensively used in oncology [3, 6, 10, 11]. However, if textural features are to be used in clinical practice, they have to be robust under the typical variations found between different scanners, acquisition protocols, resolutions, etc [12].

The influence of the acquisition protocol on textural measures has been controversial in the literature and may depend on the specific parameter, tumors studied, etc [13, 14, 15, 16, 17]. Mayerhoefer et al. [13] and Waugh et al. [14] studied the influence of different clinical breast MRI protocols and parameters on the results of several textural features. They showed that spatial resolution is the most important factor influencing textural measures, while changes to other protocol parameters did not change the outcome of texture analyses so significantly. Collewet et al. [15] studied the effects of two MRI acquisition protocols and four image intensity normalization methods for texture classification. They found that the dynamic range discretization is also important for classification, as one of the four methods considered performed significantly better than the others. Leijenaar et al. [16] studied the variability of textural features in FDG PET images due to different acquisition modes and reconstruction parameters. Finally, Molina et al [17] found that many two-dimensional textural features were not robust under the combined effect of matrix size and dynamic range variation for a small set of patients.

To our knowledge most previous work has been focused on two-dimensional (2D) descriptors. However, it is already acknowledged that three-dimensional (3D) features are more representative of tumor properties and should be used instead [2, 18–20].

3D textural features have been less used in brain tumor imaging. Two studies have depicted their discrimination properties among VOIs containing metastases, gliomas and meningiomas [21]. Only a study has proven an increased performance of 3-D GLCMs compared to 2D in tumoral tissues classification [22].

The aim of this study was to analyze the robustness of the most common second order textural features used to characterize brain tumors in three-dimensional (3D) scenarios under changes of spatial resolution and dynamic range.

## Materials and methods

### Patients

67 patients with biopsy-proven glioblastoma were first considered for the study. The inclusion criterion was the availability of 3D pretreatment T1-weighted images. After that, in order to make sound analyses, we selected the most common image configuration and exclude the remaining patients. So, 20 patients (64.80 ± 9.12 years-old) were finally included in the study, that is, those with matrix of 432x432 pixels and voxel size of 0.5x0.5x1 mm^3^ (47 patients were finally excluded).

The study was approved by the Institutional Review Board (IRB) of Hospital Universitario Marqués de Valdecilla. Informed consent was waived by the IRB because we included only patients who had previously provided authorization for use of their medical records for research.

### Imaging protocol

All examinations were performed with a three-dimensional (3D) spoiled gradient recalled echo (SPGR). T1-weighted images of the whole brain without magnetization transfer were recorded after intravenous administration of a single-dose of gadobenate dimeglumine (0.1 mmol/kg, MultiHance, Bracco; Milan, Italy) with a 6-min delay.

Images were obtained using a 3-T magnet machine (Achieva, Philips Healthcare, Best, The Netherlands) with 22 cm field of view. Imaging parameters were: repetition time/echo time (TR/TE) of 20/11 ms; flip angle of 25°; matrix 432x432 pixels, voxel size (0.5x0.5x1 mm^3^).

### Image analysis

DICOM files were imported into the scientific software package Matlab (R2015b, The MathWorks, Inc., Natick, MA, USA) and processed using an in-house semi-automatic 3D image segmentation procedure [23]. Afterwards, segmented tumors were manually corrected.

### Textural analysis

A set of textural quantities derived from co-occurrence (CM) and run-length matrices (RLM) were chosen for this study and computed for tumors after segmentation.

The CM is a square matrix with dimension equal to the number of grey levels present in the image (dynamic range). It measures the relations between pairs of voxels within an image (8). It is usually characterized by a distance between pixels (e.g. adjacent pixels, pixels having at least one common neighbor, etc.) and one angle/direction (0°, 45°, 90°, 135°). Changing the distance or the direction leads to different matrices. Many previous studies have constructed different co-occurrence matrices by combining these parameters. However, in oncological imaging a more straightforward workflow is to obtain an averaged CM matrix considering every possible direction, i.e. the relations between each voxel and its 26 neighbors in 3D [20, 24]. In this work only the 3D CM was developed, providing more robust measurements. Also, we considered unit distance to construct our CM. Thus, each cell CM(i,j) in the CM matrix corresponds to the number times that one voxel of intensity *i* is a neighbor of another voxel with intensity *j* in 3D. Most common features derived from the CM were the Entropy, Homogeneity, Contrast, Dissimilarity and Uniformity [7, 25]. For a comprehensive description of the meaning of each of the CM-based measures see Kurani et al [26].

The RLM matrix constructed in this work follows the same principles used for the CM. Thus, each cell RLM(i,j) was constructed as the number of runs of length j formed by voxels of intensity in box i considering all the 13 possible 3D directions [24, 27]. It is interesting to point out that this kind of textural analysis allows for a better characterization of tumor heterogeneity, being able to describe complex 3D structures labeled with the same grey level values. Most popular textural features based on the RLM were the Long Run Emphasis (LRE), Short Run Emphasis (SRE), Low Grey level Run Emphasis (LGRE), High Grey level Run Emphasis (HGRE), Short Run Low Grey level Emphasis (SRLGE), Short Run High Grey level Emphasis (SRHGE), Long Run Low Grey level Emphasis (LRLGE), Long Run High Grey level Emphasis (LRHGE), Grey level Non-Uniformity (LRHGE), Grey level Non-Uniformity (GLNU), Run-Length Non-Uniformity (RLNU) and Run Percentage (RPC) [27]. An intuitive description of the meaning of the RLM measures considered was done by Xu et al [28].

Further details on the measures definition can be found in Table 1.

**Table 1:** Textural features considered in this study.

Type of measure	Name	Formula
Co-occurrence matrix	Entropy	$-\sum_{i=1}^{N}\sum_{j=1}^{N}CM(i,j)\cdot \text{ln}\left[CM(i,j)\right]$
Co-occurrence matrix	Homogeneity	$\sum_{i=1}^{N}\sum_{j=1}^{N}\frac{CM(i,j)}{1+{\left(i-j\right)}^{2}}$
Co-occurrence matrix	Contrast	$\sum_{i=1}^{N}\sum_{j=1}^{N}CM(i,j)\cdot {\left(i-j\right)}^{2}$
Co-occurrence matrix	Dissimilarity	$\sum_{i=1}^{N}\sum_{j=1}^{N}CM(i,j)\cdot \left|i-j\right|$
Co-occurrence matrix	Uniformity	$\sum_{i=1}^{N}\sum_{j=1}^{N}{\left[CM(i,j)\right]}^{2}$
Run-length matrix	Long Run Emphasis (LRE)	$\frac{1}{n_{r}}\sum_{i=1}^{N}\sum_{j=1}^{M}RLM(i,j)\cdot j^{2}$
Run-length matrix	Short Run Emphasis (SRE)	$\frac{1}{n_{r}}\sum_{i=1}^{N}\sum_{j=1}^{M}\frac{RLM(i,j)}{j^{2}}$
Run-length matrix	Low Grey-level Run Emphasis (LGRE)	$\frac{1}{n_{r}}\sum_{i=1}^{N}\sum_{j=1}^{M}\frac{RLM(i,j)}{i^{2}}$
Run-length matrix	High Grey-level Run Emphasis (HGRE)	$\frac{1}{n_{r}}\sum_{i=1}^{N}\sum_{j=1}^{M}RLM(i,j)\cdot i^{2}$
Run-length matrix	Short Run Low Grey-level Emphasis (SRLGE)	$\frac{1}{n_{r}}\sum_{i=1}^{N}\sum_{j=1}^{M}\frac{RLM(i,j)}{i^{2}\cdot j^{2}}$
Run-length matrix	Short Run High Grey-level Emphasis (SRHGE)	$\frac{1}{n_{r}}\sum_{i=1}^{N}\sum_{j=1}^{M}\frac{RLM(i,j)\cdot i^{2}}{j^{2}}$
Run-length matrix	Long Run Low Grey-level Emphasis (LRLGE)	$\frac{1}{n_{r}}\sum_{i=1}^{N}\sum_{j=1}^{M}\frac{RLM(i,j)\cdot j^{2}}{i^{2}}$
Run-length matrix	Long Run High Grey-level Emphasis (LRHGE)	$\frac{1}{n_{r}}\sum_{i=1}^{N}\sum_{j=1}^{M}RLM(i,j)\cdot i^{2}\cdot j^{2}$
Run-length matrix	Grey-Level Non-Uniformity (GLNU)	$\frac{1}{n_{r}}\sum_{i=1}^{N}{\left(\sum_{j=1}^{M}RLM(i,j)\right)}^{2}$
Run-length matrix	Run-Length Non-Uniformity (RLNU)	$\frac{1}{n_{r}}\sum_{j=1}^{M}{\left(\sum_{i=1}^{N}RLM(i,j)\right)}^{2}$
Run-length matrix	Run Percentage (RPC)	$\frac{n_{r}}{\sum_{i=1}^{N}\sum_{j=1}^{M}RLM(i,j)\cdot j}$

Definition of the textural features considered in this study. For co-occurrence (CM) measures CM(i,j) stands for the co-occurrence matrix, N is the number of classes of grey-levels taken (8, 16, 32 and 64 in this study). For run-length matrix (RLM) measures RLM(i,j) is the run-length matrix, n_r_ is the number of runs, N is the number of classes of grey-levels and M is the size in voxels of the largest region found.

The construction of the CM and RLM depends on the spatial resolution and dynamic range considered. Two different matrix sizes were used in this work: 432x432 (raw matrix) and 256x256 (standard MRI matrix size). The latter was obtained by interpolation using the Matlab software (v. R2016b) on the raw images. Also, we considered two different resolutions along z-axis: 1 mm (raw) and 2 mm (also obtained by interpolation on each pair of initial slices). For these four combinations of spatial resolutions, we discretized the dynamic range in 16, 32 and 64 different grey levels, as these are the most used and recommended dynamic ranges for these measures [17]. This procedure led to 12 different datasets for each patient.

**Fig 1:**
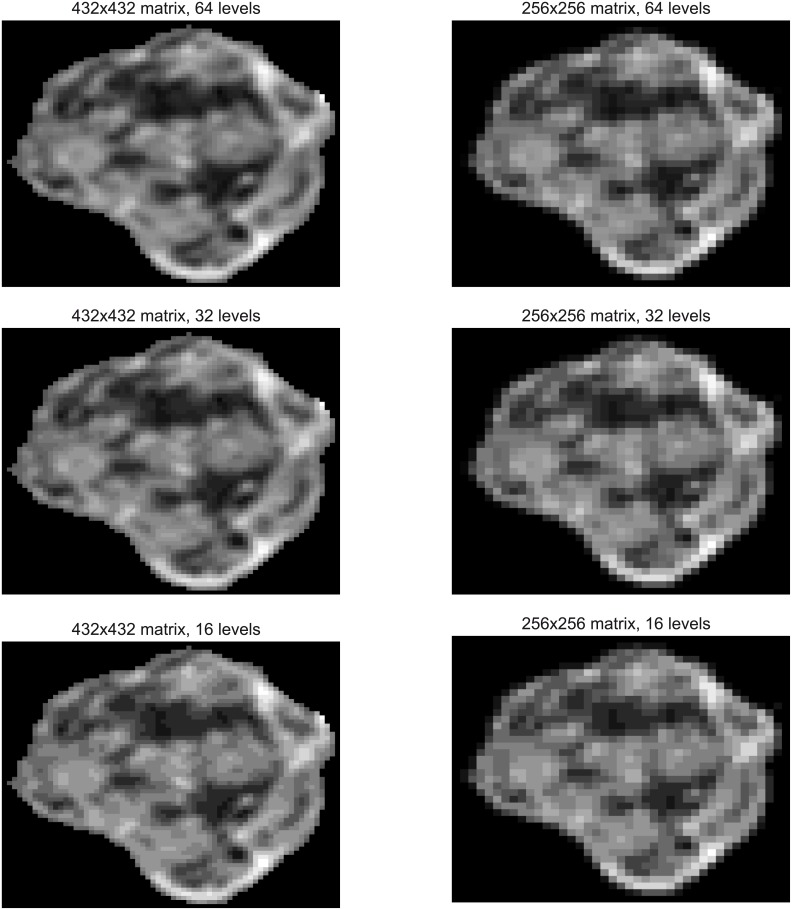
Resized and discretized images for the same slice using 432x432 and 256x256 as matrix sizes and 64, 32 and 16 as dynamic ranges. Running title of each image identifies the matrix size and dynamic range considered.

Fig 1 shows an example of six resized and discretized slices obtained from the same tumor, using 432x432, 256x256 as matrix sizes and 16, 32 and 64 as dynamic ranges.

Finally, a set of 5 CM and 11 RLM heterogeneity textural features were computed for each image configuration using the Matlab software. Table 1 shows the mathematical expression of each textural parameter computed in this study.

### Robustness evaluation

Once the textural features were computed, robustness was assessed by means of the coefficient of variation (CV) [29]. The CV is a standardized measure of dispersion which is defined as the ratio between the standard deviation and the mean of a series of data. The result is typically reported as a percentage.

The mean of the CVs was obtained for all patients included. Textural features with a mean CV smaller than 10% were considered to be robust.

## Results

We computed the features listed in Table 1 for each of the 20 patients and each combination of spatial resolution and dynamic range considered leading to a total of 3840 textural feature values. With these data we performed several comparisons, which are discussed below.

### Experiment 1. Consistency of spatial resolutions for varying dynamic ranges

For each textural feature, patient and spatial resolution, we computed the CV using data for dynamic ranges of 64, 32 and 16 grey levels. This parameter provided a measure of the dependence of the textural features under changes of the grey levels. Then, the mean value and the standard deviation for the set of 20 patients included in the study were computed.

Table 2 shows the results for the mean values obtained for the whole set of patients and the standard deviations, both in percentages. No measure was found to be robust (mean CV smaller than 10%) under the variation of the dynamic range.

**Table 2:** Mean (and standard deviation) of the CV computed for the 20 patients. Results are shown for each combination of spatial resolution and slice thickness considered. CV was computed for each feature considering different dynamic range values, i.e. 16, 32 and 64 grey levels.

		Matrix size and slice thickness			
		432x432; 1 mm	432x432; 2 mm	256x256; 1 mm	256x256; 2 mm
**Co-occurrence matrix**	**Entropy**	34.66 (6.42)	32.74 (5.04)	32.12 (5.65)	31.67 (5.23)
	**Homogeneity**	24.06 (3.99)	25.63 (3.95)	29.57 (4.12)	31.07 (4.43)
	**Contrast**	105.68 (5.25)	108.84 (3.25)	111.29 (1.52)	111.55 (1.49)
	**Dissimilarity**	66.17 (3.20)	65.80 (1.65)	66.50 (2.22)	66.50 (1.85)
	**Uniformity**	99.20 (9.25)	99.86 (10.01)	101.49 (9.82)	102.28 (10.38)
**Run-length matrix**	**LRE**	152.94 (11.34)	157.68 (10.91)	155.25 (9.34)	159.50 (9.92)
	**SRE**	13.11 (23.75)	15.16 (15.16)	12.89 (5.88)	23.41 (16.33)
	**LGRE**	94.26 (18.56)	105.25 (16.39)	42.29 (7.67)	40.59 (10.22)
	**HGRE**	102.63 (3.90)	99.27 (7.59)	100.92 (13.64)	93.95 (13.49)
	**SRLGE**	89.95 (27.58)	107.05 (16.48)	37.83 (11.03)	33.87 (15.84)
	**SRHGE**	108.95 (11.45)	108.42 (6.74)	109.96 (11.38)	112.03 (9.86)
	**LRLGE**	164.12 (7.60)	165.56 (7.45)	166.29 (4.53)	167.17 (5.03)
	**LRHGE**	114.83 (20.20)	126.48 (22.77)	113.47 (21.80)	126.18 (21.52)
	**GLNU**	36.25 (11.63)	45.63 (13.87)	38.77 (17.15)	56.06 (18.69)
	**RLNU**	84.41 (17.07)	98.13 (6.35)	99.18 (12.08)	110.37 (13.24)
	**RPC**	81.75 (9.55)	86.91 (10.91)	84.37 (12.74)	91.91 (15.21)

**Fig 2:**
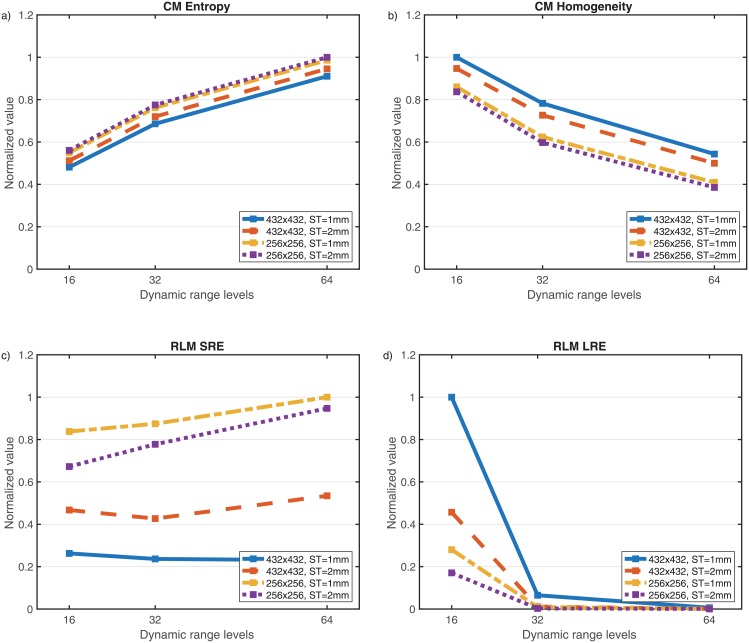
Values of several textural features (normalized to the maximum value obtained in each subplot) for different spatial resolutions (432x432 ST 1mm, 432x432 ST 2 mm, 256x256 ST 1 mm, 256x256 ST 2 mm) and dynamic range values (16, 32 and 64 grey levels). Shown are results for a) co-occurence (CM) Entropy, b) CM Homogeneity, c) run-length matrix (RLM) SRE, d) RLM LRE.

The same result was found when patients were considered individually instead of the means. For example, in Fig 2 we plot the normalized values of four relevant textural measures for a specific patient and different spatial resolutions showing their wide range of variation as a function of the dynamic range.

### Experiment 2. Consistency of textural features and dynamic ranges for varying spatial resolutions

Next, for each textural feature, patient and dynamic range, we computed the CV for the four spatial resolutions considered. The CM entropy was the outstanding feature and the only robust feature for every dynamic range observed (Table 3). Only a low dynamic range (16-gray level) homogeneity was also robust.

**Table 3:** Mean (and standard deviation) of the CV of the 20 patients’ regarding each dynamic range considered. CV was computed for each feature considering different combinations of matrix size and slice thickness, that is, matrix sizes of 432x432 and 256x256 pixels and slice thickness of 1 mm and 2 mm. Shaded cells correspond to those combinations obtaining a CV below 10%.

		Dynamic range		
		16 levels	32 levels	64 levels
**Co-occurrence matrix**	**Entropy**	7.91 (3.77)	6.31 (2.80)	5.08 (2.31)
	**Homogeneity**	7.13 (2.43)	10.89 (3.45)	13.96 (4.25)
	**Contrast**	38.94 (6.14)	44.40 (7.07)	46.86 (7.54)
	**Dissimilarity**	25.65 (5.15)	25.53 (4.79)	25.89 (4.97)
	**Uniformity**	19.54 (8.71)	21.94 (10.11)	23.15 (10.24)
**Run-length matrix**	**LRE**	108.66 (23.59)	125.03 (17.88)	117.39 (15.24)
	**SRE**	55.06 (28.39)	54.62 (14.88)	56.25 (14.71)
	**LGRE**	37.11 (6.70)	63.38 (12.02)	80.97 (11.96)
	**HGRE**	21.06 (5.48)	33.53 (6.34)	32.39 (6.40)
	**SRLGE**	65.82 (13.17)	85.34 (11.65)	97.96 (8.49)
	**SRHGE**	47.17 (34.13)	37.49 (18.52)	40.76 (15.56)
	**LRLGE**	90.24 (25.33)	107.14 (26.26)	89.65 (29.62)
	**LRHGE**	117.18 (20.80)	130.42 (14.57)	123.10 (12.52)
	**GLNU**	33.43 (7.44)	30.67 (12.23)	24.34 (7.24)
	**RLNU**	77.45 (23.39)	83.84 (15.30)	81.46 (13.09)
	**RPC**	44.25 (8.36)	58.03 (8.57)	58.12 (10.24)

**Fig 3:**
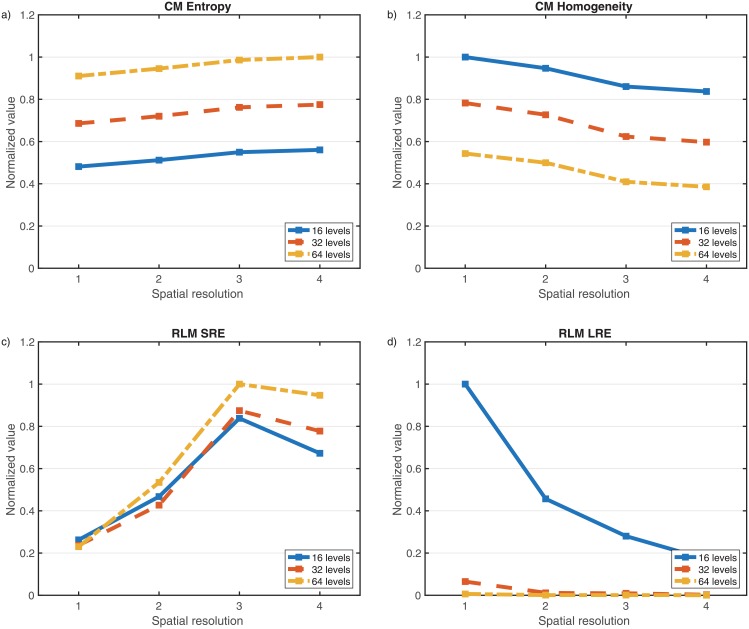
Values of several textural features (normalized to the maximum value obtained in each subplot) for different dynamic range values (16, 32 and 64 grey levels) and spatial resolutions (432x432 ST 1mm, 432x432 ST 2 mm, 256x256 ST 1 mm, 256x256 ST 2 mm). Shown are results for a) CM Entropy, b) CM Homogeneity, c) RLM SRE, d) RLM LRE.

Textural features dependence of different image reconstruction parameters is shown in Fig 3.

## Discussion

The analysis of texture parameters is a useful way of increasing the information obtainable from medical images.

This is a promising field of research, with applications ranging from the detection of lesions to differentiation between pathological and healthy tissue in different organs and the segmentation of anatomical structures. Texture analysis uses radiological images obtained in routine diagnostic practice, but involves an ensemble of mathematical computations performed with the data contained within the images. [1]

In oncology, there is an increasing evidence that texture analysis has the potential to augment diagnosis and characterization as well as improve tumor staging and therapy response assessment in oncological practice [2].

Different textural features, tumors and imaging modalities have been considered in the literature for cancer detection [5, 6], response to treatment [30] and survival group prediction [31]. However, the robustness of these methods under changes on 3D spatial resolution and dynamic range has not received much attention in brain MR imaging, being more popular in PET imaging [16, 32, 33]. In our study, none of the measures was robust under dynamic range changes, as it has been observed in limited 2D studies [17]. Thus, even at high spatial resolution, dynamic range substantially influences the textural measures' results.

Several works have employed CM and RLM based 2D features, using varied distances and directions between pixels, leading to large sets of ‘different’ measures [25, 29, 30]. However, in brain tumor imaging this workflow is meaningless, since patients are not explored exactly in the same positions, so different pixel distributions are compared using this methodology. Also, tumors are 3D structures, where study of their parameters in all the 3D directions is encouraged and would lead to a better characterization of tumor heterogeneity [20]. Reproducibility and robustness assessment are instigated in all fields of imaging and radiomics [34].

Previous findings imply, as long textural features should be used in clinical practice, that dynamic range has to be fixed in order to compare different studies. Its optimal threshold depends on the requisites of imaging analysis [16]. A dynamic range of 8 grey levels has been discouraged due to an insufficient contrast range resolution [35]. On the other hand, if the dynamic range is too large, many close grey-level values would be separated into different regions. Thus, we recommend acquiring either 16 or 32 different grey levels as dynamic range.

In any case, the very concept of identification of spatial structures by their closeness in an arbitrary ‘uniform’ grey level scale is one of the major weaknesses of RLM-based features, whatever is the number of grey levels taken.

As to the spatial resolution, we used only high resolution brain tumor scans in line with recent recommendations [36]. Thus, inconsistency of textural parameters is due to their lack of robustness.

Only the CM entropy was robust under spatial variations in our analysis. This feature is one of the most relevant textural measures and has been reported to have a predictive value in GBM patients [37], to distinguish radiation necrosis from metastasis in brain non-small cell lung cancer oligometastasis [38]. In other imaging settings, CM entropy was able to differentiate among breast cancer subtypes [39], in non-small cell lung cancers undergone to concomitant chemoradiotherapy [40] and between malignant and inflammatory pulmonary nodules [41]. In spite of its relevance, the fact that the remaining 15 measures studied were not robust, focus on their self-limitations as image complexity descriptors. These shortcomings are shared with PET textural analysis [32].

One would expect properly defined measures to be only weakly dependent–or no dependent at all- on the spatial discretization parameters. At least, they should converge to certain limit values when both the dynamic range and/or the spatial resolutions are sufficiently high. This is not the case for the measures studied here. We choose the RLM and CM measures because of their widespread use in oncological image-based studies either as individual predictors or as ‘imaging genes’ in the context of radiomics.

On the basis of our results there is a need for standardization of the spatial resolution if these measures are to be used in clinical practice. Most current images can be registered at a matrix size of 256x256. As to the slice thickness it should be fixed to 1 mm with 0 mm gap. This provides typically voxels of volume around 1 mm^3^ [36].

This study has some advantages over previous works. First, to the best of our knowledge, robustness analysis under spatial resolution and grey-level changes has not been performed in brain tumor MR imaging. Secondly, our study used standard MR contrast-enhanced T1-weighted sequences (CE-T1WI). Also, we used normalized textural measures definitions [32].

As to the potential limitations of our study, we have to point out the synthetic nature of our methodology: we decided to use one T1 sequence and use the Matlab software to generate three additional sequences from it. Another option could have been to submit patients to four different MRIs with different image parameters. However, that procedure would be susceptible to patient’s movement artifacts, contrast-enhancement decay along time or other external variations. On the other hand, interpolation methods have shown their effectiveness, thus not changing substantially image information and reducing such possible artifacts [42]. Second, we did not compare T1-weighted sequences among different vendors. It may happen that early assumptions of good compatibility in textural analysis in multicenter studies irrespective of the scanner and acquisition parameters were too optimistic [43, 44]. Another limitation is that we only dealt with certain textural features, but we choose the most meaningful ones that are also those more used in the literature. Also, we only showed the robustness of CE-T1WI sequence, and other imaging sequences could be also analyzed [45].

In this work, the variations of textural features due to different imaging protocols using postcontrast pretreatment 3D CE-T1WI were assessed. The displayed lack of robustness lies in the nature of the measures considered and not on the specific sequence, however it could be interesting to analyze the variability of the measures using other imaging sequences such as 3D FLAIR.

The results found here go beyond of neuro-oncology setting and suggest that textural feature analysis of oncological images should be done carefully if results from different scanners/resolutions/dynamic ranges are to be compared. Also, while there are other types of textural features, such as those of spectral type [1, 10], most used parameters share such limitations. This fact raises the need for the development of additional families of texture descriptors, less dependent on matrix size and/or dynamic range.

## Conclusions

We have performed a wide analysis of the robustness of usual 3D textural features regarding different matrix sizes and dynamic range configurations. Results achieved show that 3D textural feature values depend substantially on the dynamic range, thus it is mandatory to fix it in order to obtain reliable and comparable results. After fixing the dynamic range, the CM Entropy was the only robust textural measure under spatial discretization changes. Due to the lack of robustness of the other measures, their use to assess heterogeneity in multi-center studies has to be standardized. As practical recommendations for brain tumor images we recommend using 16 levels of gray as dynamic range and resampling higher resolution images to 256x256 as matrix size corresponding typically to 1 mm voxels.
